# Characteristic Alterations of Network in Patients With Intraoperative Stimulation-Induced Seizures During Awake Craniotomy

**DOI:** 10.3389/fneur.2021.602716

**Published:** 2021-03-18

**Authors:** Shengyu Fang, Chunyao Zhou, Lei Wang, Xing Fan, Yinyan Wang, Zhong Zhang, Tao Jiang

**Affiliations:** ^1^Department of Neurosurgery, Beijing Tiantan Hospital, Capital Medical University, Beijing, China; ^2^Beijing Neurosurgical Institute, Capital Medical University, Beijing, China; ^3^Research Unit of Accurate Diagnosis, Treatment, and Translational Medicine of Brain Tumors Chinese (2019RU11), Chinese Academy of Medical Sciences, Beijing, China

**Keywords:** intraoperative stimulation-induced epilepsy, direct cortical stimulating, glioma, seizure, sensorimotor network

## Abstract

**Background:** The use of electrocorticography (ECoG) to avoid intraoperative stimulation-induced seizure (ISS) during awake craniotomy is controversial. Although a standard direct cortical stimulating (DCS) protocol is used to identify the eloquent cortices and subcortical structures, ISS still occurs. Epilepsy is related to alterations in brain networks. In this study, we investigated specific alterations in brain networks in patients with ISS.

**Methods:** Twenty-seven patients with glioma were enrolled and categorized into the ISS and non-ISS groups based on their history of ISS occurrence. A standard DCS protocol was used during awake craniotomy without ECoG supervision. Graph theoretical measurement was used to analyze resting-state functional magnetic resonance imaging data to quantitatively reveal alterations in the functional networks.

**Results:** In the sensorimotor networks, the glioma significantly decreased the functional connectivity (FC) of four edges in the ISS group, which were conversely increased in the non-ISS group after multiple corrections (*p* < 0.001, threshold of *p*-value = 0.002). Regarding the topological properties, the sensorimotor network of all participants was classified as a small-world network. Glioma significantly increased global efficiency, nodal efficiency, and the sigma value, as well as decreased the shortest path length in the ISS group compared with the non-ISS group (*p* < 0.05).

**Conclusions:** The specific alterations indicating patient susceptibility to ISS during DCS increased global and nodal efficiencies and decreased the shortest path length and FC induced by gliomas. If the patient has these specific alterations, ECoG is recommended to monitor after-discharge current during DCS to avoid ISS.

## Introduction

Awake craniotomy (AC) is the optimal approach to prevent neurological deficits when eloquent structures are invaded (1). Intraoperative stimulation-induced seizure (ISS) is the most serious complication of AC. Despite the application of a standard protocol of direct cortical stimulation (DCS) and using ice-cold Ringer's solution for control (2, 3), ISS is difficult to prevent (incidence was 2.2–21.5%) (1, 4–14). If ISS occurs, patients will have poor cooperation, and the time of functional monitoring will be prolonged, and the accuracy of identifying functional areas will be decreased. Accordingly, ISS prevention is crucial in AC.

Electrocorticography (ECoG) is a minimally invasive technique of intraoperative neuro-monitoring. Whether or not ECoG should be used in AC to prevent ISS remains controversial (2, 4). Some traditional studies supported the use of ECoG during AC to establish the threshold of stimulation and capture after-discharges (15). In contrast, Boetto et al. (16) suggested that ECoG was unnecessary for AC. They proposed that ISS incidence was only 3.5% and that using ECoG would only complicate the surgical process. Hence, identifying patients susceptible to ISS is important to clarify the standards for using ECoG.

Seizures are considered related to brain network alterations. However, the association between ISS and alterations in functional networks remains poorly understood. Resting-state functional magnetic resonance imaging (rs-fMRI) with graph theoretical analyses can reveal the distribution of brain networks and identify changes in topological properties. Hence, we enrolled glioma patients who underwent AC and performed rs-fMRI analysis using a graph theoretical approach. We aimed to find characteristic differences in functional networks between patients with and without ISS and to ascertain which patients are suitable for ECoG to capture after-discharges during AC.

## Materials and Methods

The institutional review board of Beijing Tiantan Hospital approved this study. All enrolled patients and participants provided written informed consent.

### Participants

We retrospectively reviewed 84 patients diagnosed with gliomas who underwent AC with DCS to preserve motor, sensory, and motor-related language functions between March 2017 and March 2019 at Beijing Tiantan hospital. The inclusion criteria were as follows: (a) patient aged >18 years; (b) no history of biopsy, radiotherapy, or chemotherapy. The exclusion criteria were as follows: (a) contraindications for MRI; (b) head motion >1 mm in translation or 1° in rotation; and (c) the administration of antiepileptic drugs before preoperative rs-fMRI scanning. All enrolled patients were classified into epileptic (ISS) and non-epileptic (non-ISS) groups based on ISS occurrence. Moreover, all patients received 0.5 g levetiracetam twice a day to prevent glioma-related epilepsy after rs-fMRI scanning. We also recruited 20 healthy participants matched for age, sex, and education level.

### Clinical Characteristics

Data for age, sex, education level, preoperative epilepsy, Karnofsky Performance Scale (KPS) score, and histopathology were derived from inpatient records and results of preoperative electroencephalograms. ISS and stimulation current information was derived from surgical records and intraoperative photos.

### Intraoperative Stimulation Protocol

The stimulation protocol was the same as that described previously (17). The Ojemann stimulators with 5-mm diameter were employed to identify the eloquent cortices (intensity, 1–6 mA; frequency, 60 Hz; square wave). The stimulation current began at 1 mA and was increased by 0.5-mA increments until the stimulation threshold was established. The stimulation threshold was determined by inducing unconscious movements (precentral gyrus stimulation) or transient numbness (postcentral gyrus stimulation). If the stimulation threshold were established, the stimulation current would remain constant and would be used to identify the eloquent cortices and subcortical structures. The duration of stimulation for identification was 1 s for sensorimotor-related structures and 4 s for language-related structures. No site was continuously stimulated. Whenever ISS occurred, ice-cold Ringer's solution was used to terminate it. If the seizure duration was over 10 s, benzodiazepine medications were administered, and functional monitoring was discontinued. ECoG was not used to capture after-discharges or determine the stimulation threshold.

### MRI Acquisition

A MAGNETOM Prisma 3T MR scanner (Siemens, Erlangen, Germany) was used to acquire all image data. The parameters of MRI sequences (T1, FLAIR, and rs-fMRI) were as follows: T1 magnetization-prepared rapid acquisition gradient echo with gadolinium enhancement to acquire anatomical images, repetition time (TR): 2,300 ms; echo time (TE): 2.3 ms; field of view (FOV): 240 × 240 mm^2^; flip angle: 8°; slice number: 192; voxel size: 1.0 mm^3^ × 1.0 mm^3^ × 1.0 mm^3^; FLAIR sequence, TR: 5,000 ms; TE: 387 ms; FOV: 220 mm^2^ × 220 mm^2^; flip angle: 150°; slice number: 128; thickness: 0.9 mm; voxel size in panel: 0.4 mm^3^ × 0.4 mm^3^ × 0.9 mm^3^; rs-fMRI sequence, TR: 2,000 ms; TE: 30 ms; FOV: 220 mm^2^ × 220 mm^2^; flip angle: 90°; slice number: 30; voxel size in panel: 3.0 mm^3^ × 3.0 mm^3^ × 3.0 mm^3^, acquisition duration: 8 min.

Participants were asked to close their eyes without thinking about anything in particular during rs-fMRI acquisition.

### Regions of Tumor Invasion

The MRIcron software (http://www.mccauslandcenter.sc.edu/mricro/mricron/) was used to manually draw the extent of glioma invasion (shown in Supplementary Figure 1), in the individual patient images, by two neuroradiologists (with 10 years' experiences of glioma diagnosis) independently based on the enhanced regions of the FLAIR images for low-grade glioma and T1 enhancement images for high-grade glioma. If there was over 5% difference in the region between the two drawn images, a third neuroradiologist with 20 years' experience made the final decision. The drawn regions of tumor invasion of all patients were normalized into the MNI-152 T1 template using SPM 8 (University College London, London, United Kingdom; http://www.fil.ion.ucl.ac.uk/spm/). The tumor volume was calculated using MRIcron software.

### Functional MRI Preprocessing

A graph theoretical network analysis toolbox (GRETNA, https://www.nitrc.org/projects/gretna) (18, 19) was used for rs-fMRI preprocessing. The pipeline of preprocessing was as follows (20): (a) data transformation (from DICOM to NIFTI); (b) removal of the first 10 images; (c) timing slice; (d) realignment; (e) normalization (normalized to the EPI template) (21); (f) smoothing (full width half maximum: 6 mm); (g) linear detrending; (h) regressing out covariance [cerebrospinal fluid (CSF) signal: with CSFMask_3 mm; white matter signal: with WMMask_3 mm; head motion: Friston-24 parameters]; (i) temporal filtering (0.01–0.1 Hz); and (j) scrubbing (using default parameters according to the interpolation strategy: linear interpolation; FD threshold = 0.5; previous time point number = 1; subsequent time point number = 2).

### Regions of Interest

All ISSs occurred during the monitoring of motor reactions on the cortex. Hence, we focused on the sensorimotor network template that was extracted from a brain atlas, “brainnetome atlas” (http://www.brainnetome.org/) (22). The seeds were generated as spheres/circles of 5-mm diameter based on the coordinates of the sensorimotor network. To avoid the effects of neurovascular uncoupling or tumor involvement, the regions invaded by gliomas were excluded (Supplementary Table 1).

### Network Construction

To construct the functional connectivity (FC) matrix, Pearson's correlation coefficients were used to compare regional mean time series for all extracted nodes of sensorimotor networks.

### Graph Theoretical Measures

Graph theoretical analyses were used to calculate global and nodal topological properties [the detailed information of topological properties is shown in the Supplementary Material (Part 1)]. All matrices were transformed into absolute and binary values to calculate topological properties.

### Statistical Analyses

Clinical characteristics were compared between the patient groups using Student's *t*-test, Mann–Whitney U tests, chi-square tests, Fisher's exact tests, and one-way analysis of variance (ANOVA) according to the type of data. To explore group differences in network topological properties, we applied a series of sparsity thresholds (0.17–0.33; interval, 0.01) consistent with a previous study (23). False discovery rate (FDR) corrections were used to correct FC. Moreover, an eta-squared correlation was applied to explore the relationship between ISS and FC values. Topological properties were compared among the groups by one-way ANOVA test. Least significant difference (LSD) was subsequently used for *post-hoc* analysis when the results of one-way ANOVA were found to be significantly different between the three groups (the ISS, non-ISS, and healthy groups). A *p*-value <0.05 was considered significant.

## Results

### Demographic Characteristics

Owing to the limited number of patients with right hemispheric gliomas accompanying ISS (two patients), 27 patients with left hemispheric gliomas were finally enrolled (men, *n* = 16; all right-handed; Tables 1, 2). The screening process of recruiting patients is shown in Supplementary Figure 2. After matching for age, sex, and education level, 20 healthy participants were enrolled as controls (men, *n* = 11).

**Table 1:** Demographic and clinical characteristics.

**Demographic and** ** clinical characteristics**	**ISS group** ** (*n* = 12)**	**Non-ISS group (*n* = 15)**	**Healthy** ** (*n* = 20)**	***p*-value**
**Gender**				
Male	6	10	11	0.43
Female	6	5	9	
Age (y)^*^	41.5 ± 4.3	44.4 ± 3.0	40.7 ± 1.7	0.57
**Handedness**				
Right	12	15	20	-
Left	0	0	0	
**KPS score (pre-operative)**				
100	10	14	-	0.56
90~100	2	1	-	
**KPS score (post-operative 3 months)**				
100	10	15	-	0.18
90~100	2	0	-	
Education level (years)^*^	14.7 ± 1.0	15.2 ± 0.9	15.3 ± 0.4	0.85
**Histopathology**				
Astrocytoma	5	5	-	-
Oligodendroglioma	3	4		
Anaplastic astrocytoma	1	3		
Glioblastoma	3	3		
**IDH status**				
Mutation	6	6	-	0.78
Wild type	6	9	-	
Tumor volume (ml)^*^	29.93 ± 4.34	33.98 ± 5.29	-	0.57
Stimulation current (mA)^*^	3.3 ± 0.4	3.0 ± 0.3	-	0.69

* *Values are means ± standard error of the mean*.

*ISS group, group of patients with intraoperative stimulation-induced epilepsy*.

*Non-ISS group, group of patients without intraoperative stimulation-induced epilepsy*.

*Using Mann–Whitney U-test to compare the difference of Karnofsky performance status between the ISS and non-ISS groups*.

*Using Student's t-test to compare the difference of tumor volume and stimulation current between the ISS and non-ISS groups*.

*Using one-way ANOVA test to compare the differences of age and education level between the ISS, non-ISS, and healthy groups*.

*Using Fisher's exact test to compare the differences of gender and Iocitrate dehydrogenase status between the ISS and non-ISS groups*.

*KPS, karnofsky performance status; IDH, iocitrate dehydrogenase*.

**Table 2:** Clinical information of enrolled patients.

**Number of patients**	**Age**	**Gender**	**Tumor location**	**Tumor volume (ml)**	**Preoperative KPS score**	**Intraoperative stimulated seizure**	**Stimulation current (mA)**	**Histo-pathology**	**IDH status**
1	34	F	Inferior frontal lobe and precentral gyrus	18.8	90	No	6	GBM	Wild type
2	30	F	Inferior frontal lobe and precentral gyrus	9.20	100	Yes	6	O	Mutation
3	30	M	Postcentral gyrus	12.54	100	No	4	AA	Wild type
4	52	F	Precentral gyrus	11.26	90	Yes	3	GBM	Wild type
5	54	F	Inferior frontal lobe and precentral gyrus	13.22	100	Yes	4	A	Wild type
6	29	M	Insular lobe	40.49	100	Yes	6	A	Mutation
7	50	F	Insular lobe	59.77	100	Yes	2.5	AA	Mutation
8	64	M	Inferior frontal lobe and insular lobe	20.77	100	Yes	2.5	A	Wild type
9	19	F	Inferior frontal lobe and insular lobe	34.94	90	Yes	2	GBM	Wild type
10	39	F	Precentral gyrus	24.45	100	No	2	A	Mutation
11	53	M	Paracentral lobe	17.60	100	No	2	AA	Wild type
12	56	M	Premotor area	13.95	100	No	5.5	A	Wild type
13	44	M	SMA and premotor area	18.86	100	No	2.5	AA	Wild type
14	52	F	Postcentral gyrus	49.33	100	No	2	O	Mutation
15	40	F	Precentral gyrus	36.77	100	Yes	4	A	Wild type
16	63	M	Inferior frontal lobe and precentral gyrus	29.25	100	Yes	3	GBM	Wild type
17	35	F	SMA and premotor area	29.29	100	No	1.5	O	Mutation
18	64	M	Postcentral gyrus	43.48	100	No	3	A	Wild type
19	30	M	Inferior frontal lobe and precentral gyrus	46.04	100	Yes	2	A	Mutation
20	32	F	Postcentral gyrus	84.16	100	No	2	GBM	Wild type
21	40	F	Inferior frontal lobe and precentral gyrus	31.26	100	No	4	A	Wild type
22	31	F	Postcentral gyrus	53.29	100	No	2	GBM	Wild type
23	45	M	Inferior frontal lobe and insular lobe	34.26	100	No	3	O	Mutation
24	46	M	Postcentral gyrus	18.16	100	No	3	A	Mutation
25	39	M	Inferior frontal lobe and precentral gyrus	31.26	100	Yes	2	O	Mutation
26	65	M	Postcentral gyrus	60.26	100	No	3	O	Mutation
27	28	F	Inferior frontal lobe	26.23	100	Yes	2	O	Mutation

*KPS, karnofsky performance status; A, astrocytoma; O, oligodendroglioma; AA, anaplastic astrocytoma; GBM, glioblastoma; M, male; F, female.

No significant differences were observed in age, sex, or education level among the three groups. No differences were seen in preoperative and postoperative KPS scores, tumor volumes, stimulation current, and the proportion of patients with preoperative epilepsy between the ISS and non-ISS groups (Table 1).

### Functional Connectivity

**Figure 1:**
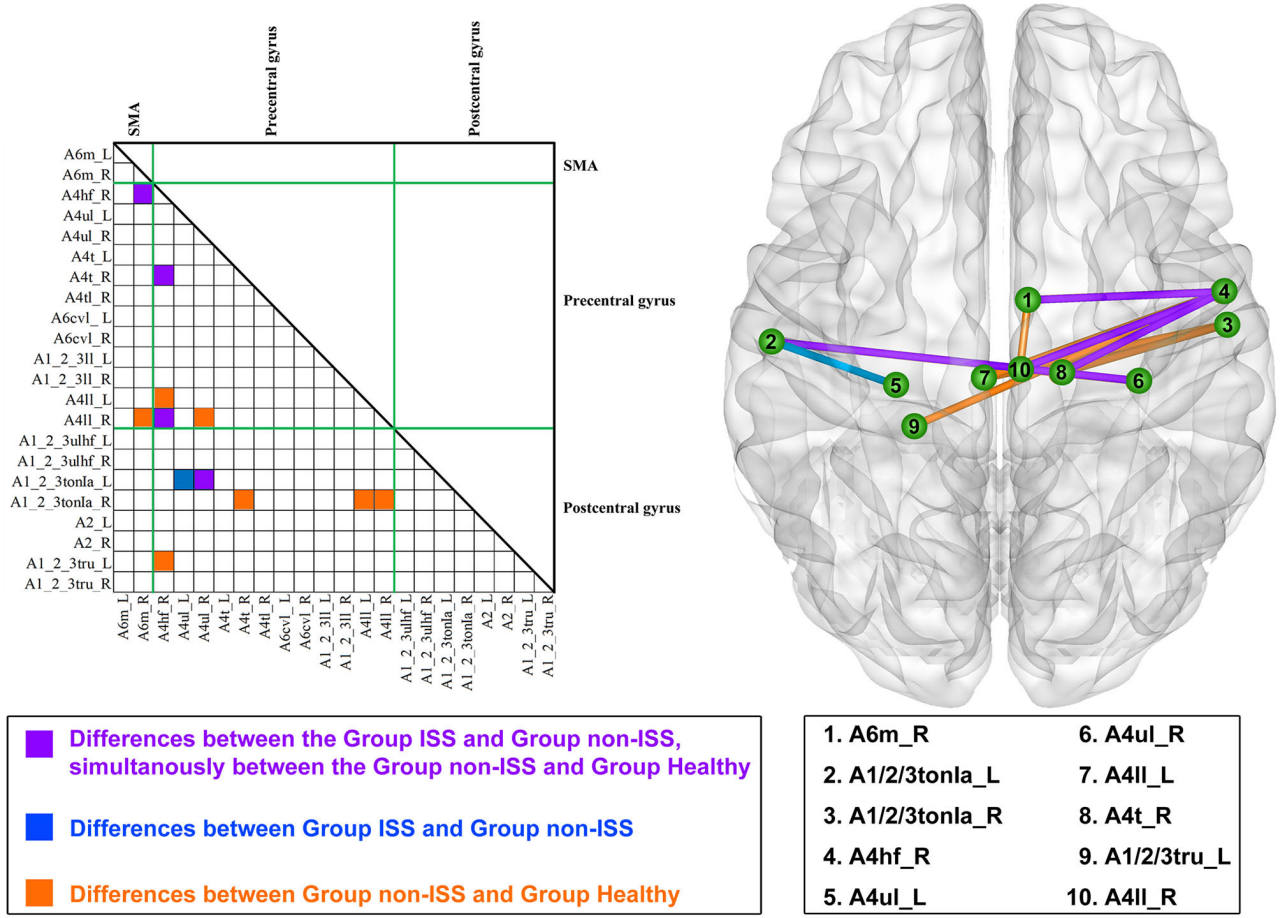
Alterations in functional connectivity (FC) among the ISS, non-ISS, and healthy groups. ISS, group of patients with intraoperative stimulation-induced seizures; Non-ISS, group of patients without intraoperative stimulation-induced seizures.

Compared with the non-ISS group, four functional edges had a decreased FC in the ISS and healthy groups after FDR correction (*p*-value threshold = 0.002; Figure 1, Supplementary Table 2) as follows: (1) the medial Brodmann area (BA) 6 in the right hemisphere (A6m_R) and BA 4 (head and face) in the right hemisphere (A4hf_R): ISS vs. non-ISS, *p* < 0.001; non-ISS vs. healthy, *p* < 0.001; (2) A4hf_R and the BA 4 (trunk) in the right hemisphere (A4t_R): ISS vs. non-ISS, *p* < 0.001; non-ISS vs. healthy, *p* < 0.001; (3) A4hf_R and the BA 4 (low limb) in the right hemisphere (A4ll_R): ISS vs. non-ISS, *p* < 0.001; non-ISS vs. healthy, *p* < 0.001; and (4) the BA 4 (upper limb) in the right hemisphere (A4ul_R) and BA 1/2/3 (tongue and larynx) in the left hemisphere (A1_2_3tonIa_L): ISS vs. non-ISS, *p* < 0.001; non-ISS vs. healthy, *p* < 0.001.

Moreover, compared with the non-ISS group, the FC of the edge that was between BA 4 (upper limb) in the left hemisphere (A4ul_L) and BA 1/2/3 (tongue and larynx) in the left hemisphere (A1_2_3tonIa_L) only decreased in the ISS group (ISS vs. non-ISS, *p* < 0.001).

Furthermore, compared with the non-ISS group, seven functional edges only decreased FC in the healthy group but were insignificantly altered in the ISS group after FDR correction. The detailed information is shown in Supplementary Table 2.

### The Relationship Between Functional Connectivity and Intraoperative Stimulation-Induced Seizure Occurrence

Four negative correlations were found between ISS occurrence and FC in four functional edges. The detailed correlations were as follows: (1) A6m_R and A4hf_R: r = −0.734, *p* < 0.001, eta-squared correlation; (2) A4hf_R and A4t_R: r = −0.696, *p* = 0.001; (3) A4hf_R and A4ll_R: *r* = −0.687, *p* = 0.001; and (4) A4ul_R and A1_2_3tonIa_L: *r* = −0.695, *p* = 0.001.

### Global Topological Properties

**Figure 2:**
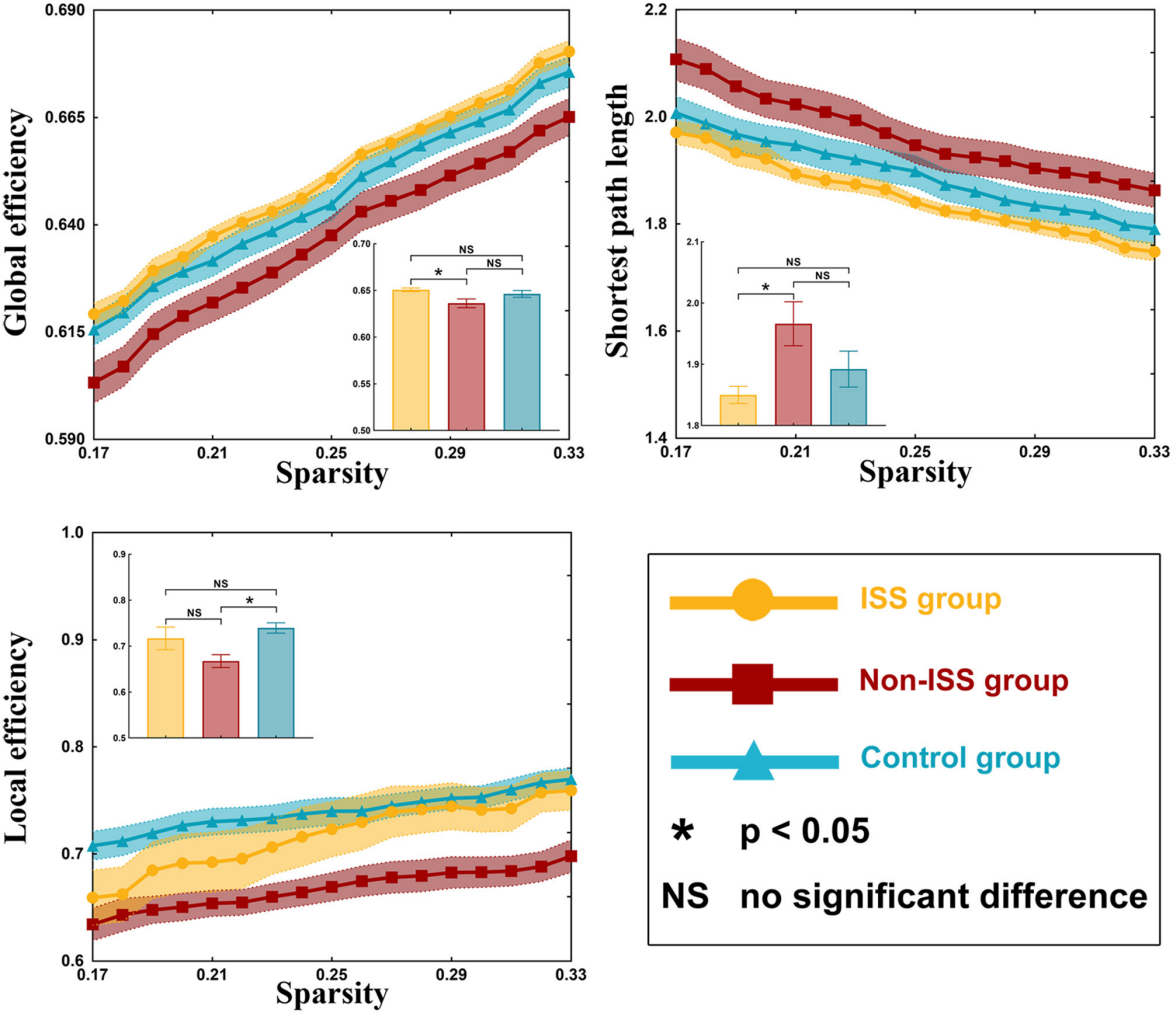
Global properties of the ISS, non-ISS, and healthy groups. ISS, group of patients with intraoperative stimulation-induced seizures; Non-ISS, group of patients without intraoperative stimulation-induced seizures.

There were some differences in global efficiency (*p* = 0.041), shortest path length (*p* = 0.043), and local efficiency (*p* = 0.009) between the three groups after one-way ANOVA (Supplementary Table 3, Figure 2).

On *post-hoc* analysis with LSD test, the non-ISS group (0.636 ± 0.004) showed weaker global efficiency than the ISS group (0.651 ± 0.002, *p* = 0.027). Moreover, compared with the non-ISS group (1.966 ± 0.034), the shortest path length was shorter in the ISS group (1.850 ± 0.013, *p* = 0.026). Additionally, compared with the non-ISS group (0.667 ± 0.013), the local efficiency was greater in the healthy group (0.740 ± 0.011, *p* = 0.002).

### Small Worldness Properties

**Figure 3:**
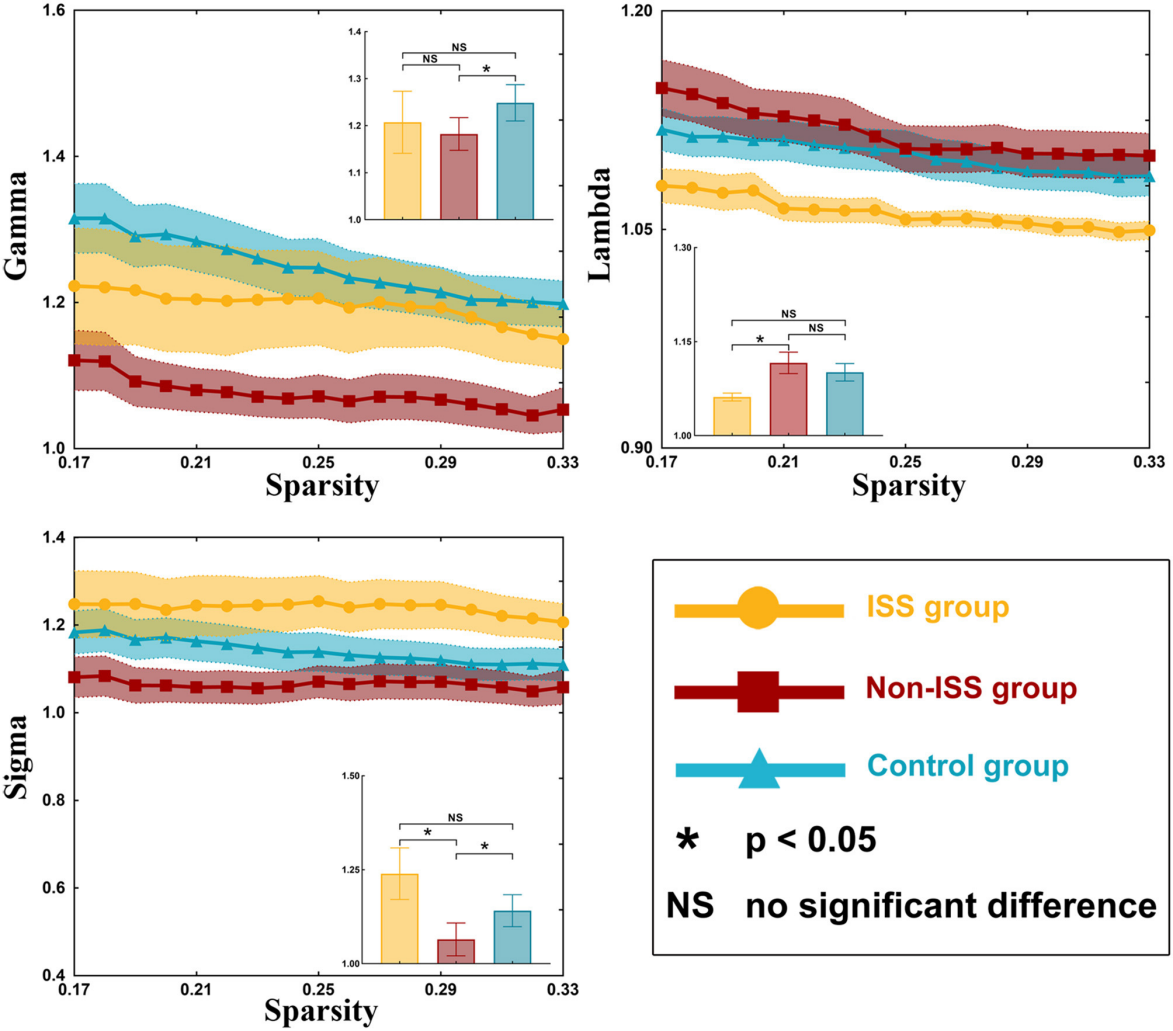
Small-world properties of the ISS, non-ISS, and healthy groups. ISS, group of patients with intraoperative stimulation-induced seizures; Non-ISS, group of patients without intraoperative stimulation-induced seizures.

Our results showed that all three groups were a small-world network because gamma value was >1 and lambda was nearly equal to 1 (γ > 1, λ ≈ 1; Supplementary Table 3). There were some differences observed in gamma value (*p* = 0.037) and sigma value (*p* = 0.046) among the three groups using one-way ANOVA (Supplementary Table 3, Figure 3).

After *post-hoc* analysis with LSD test, compared with the non-ISS group (1.075 ± 0.030), the gamma value was greater in the healthy group (1.249 ± 0.038, *p* = 0.011). Moreover, compared with the non-ISS group (0.968 ± 0.038), the sigma value was greater in the ISS (1.127 ± 0.059, *p* = 0.044) and healthy (1.14 ± 0.041, *p* = 0.017) groups.

### Nodal Topological Properties

**Figure 4:**
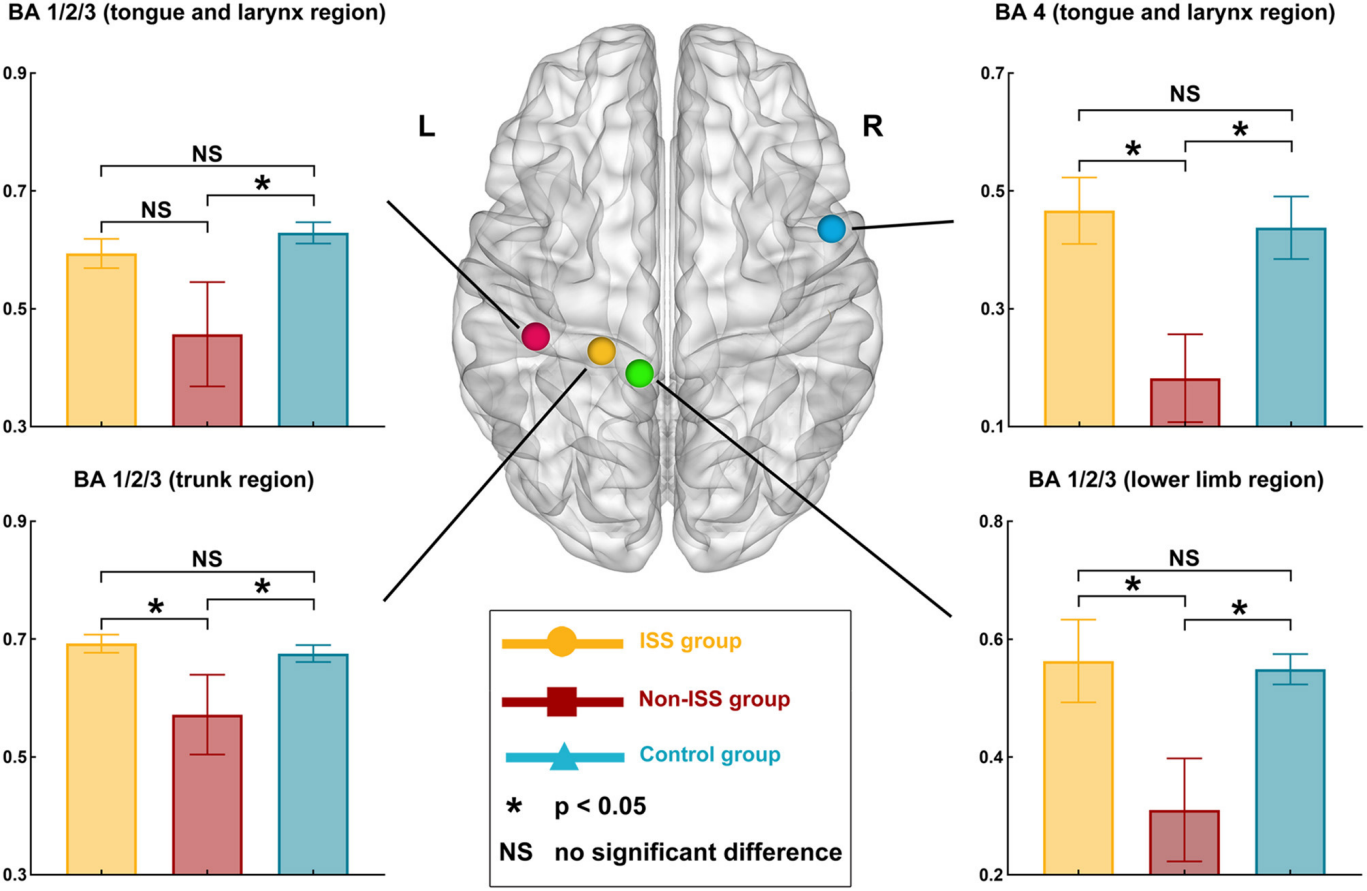
Alterations in nodal efficiency in the ISS, non-ISS, and healthy groups. ISS, group of patients with intraoperative stimulation-induced seizures; Non-ISS, group of patients without intraoperative stimulation-induced seizures.

After one-way ANOVA, there were some differences in nodal efficiency between the three groups. These nodes were BA area 4 (tongue and larynx region) in the right hemisphere (A4tl_R, *p* = 0.009), BA area 1/2/3 (lower limb region) in the left hemisphere (A1/2/3ll_L, *p* = 0.022), BA area 2 in the left hemisphere (A2_L, *p* = 0.006), and A1/2/3tru_L (*p* = 0.048) (Supplementary Table 4, Figure 4). No differences in other nodal properties (cluster efficiency, nodal shortest path length, and nodal local efficiency) were found among the three groups.

After *post-hoc* analysis with LSD test, compared with the non-ISS group (0.182 ± 0.071), the nodal efficiency of A4tl_R increased in the ISS (0.466 ± 0.054, *p* = 0.007) and healthy (0.437 ± 0.052, *p* = 0.006) groups. Moreover, compared with the non-ISS group (0.457 ± 0.084), the nodal efficiency of A1/2/3ll_L increased in the healthy group (0.629 ± 0.018, *p* = 0.007). Additionally, compared with the non-ISS group (0.310 ± 0.083), the nodal efficiency of A2_L increased in the ISS (0.563 ± 0.067, *p* = 0.006) and healthy (0.549 ± 0.025, *p* = 0.007) groups. Furthermore, compared with the non-ISS group (0.572 ± 0.064), the nodal efficiency of A1/2/3tru_L increased in the ISS (0.692 ± 0.015, *p* = 0.028) and healthy (0.675 ± 0.014, *p* = 0.029) groups.

## Discussion

This study investigated the alterations of FC and topological properties in the sensorimotor networks of patients with and without ISS during DCS. We found that the glioma induced some totally opposing alterations in FC and topological properties, thus leading to different susceptibilities to ISS in patients.

In our treatment center, ISS incidence during AC was 16.7% (14/84) using the same routine stimulation protocol as in a previous study (24). This was consistent with that reported in previous studies (2.2–21.5%) (1, 4–14).

In this study, all ISSs occurred during sensory and motor function monitoring. All sites inducing ISS were located in the sensorimotor network. Consequently, we mainly focused on alterations in the sensorimotor network.

### Functional Connectivity Alterations

Different alterations in FC were associated with ISS. Here, we found that there were four edges that decreased FC in the ISS group and simultaneously increased FC in the non-ISS group. It is widely accepted that glioma can disrupt functional networks and induce reorganization of the disrupted networks (25, 26). In our study, no patient had preoperative motor or sensory findings, indicating that sensorimotor functions of these patients were compensated. Hence, all FC alterations in the sensorimotor network were related to glioma-induced disruption and reorganization. Additionally, our findings showed that the glioma-induced reorganization was different. Such difference may lead to the stratification of susceptibility to ISS. Furthermore, our findings regarding the negative correlations between FC and occurrence of ISS revealed that the decreased FC may be a potential marker for identifying patients susceptible to ISS.

### Topological Property Alterations

Glioma-induced alterations of topological properties in ISS patients were converse to those of patients without ISS. The increased global efficiency, sigma value, and decreased shortest path length represented that the ability of information transiting is strong. Hence, these results indicated that gliomas could strengthen the ability of information conduction in ISS patients and weaken them in non-ISS patients. Moreover, a shortening network path contributes to reducing the convulsive threshold underlying epileptic seizures (27, 28). Conversely, longer network paths are related to a prolonged system response time that counteracts the rapid spread of local epileptic discharges (29). Consequently, the strengthened ability of information conduction and shortened network pathway can make the patients susceptible to ISS. Some studies focused on the temporal lobe epilepsy found that the longer shortest path length was related to seizure onset (30). We thought that this difference was due to different pathologies between idiopathic seizure and glioma-related epilepsy. The low-grade glioma grows slowly and induces network reorganization easily, and the idiopathic seizure often leads to gray matter atrophy (31) and hypometabolism (32). Hence, the decreased shortest path length was often found in patients with glioma-related epilepsy, and the increased shortest path length was often found in patients with temporal lobe epilepsy (20). Moreover, our results showed that nodal efficiency of three regions of BA 1/2/3 areas (including trunk, tongue, and lower limb) increased in the ISS group. These findings indicated that those nodes were activated to participate in the glioma-induced motion generation process in the ISS group, but those nodes were inhibited from participating in this process in the non-ISS group. The BA 1/2/3 areas were directly associated with information conduction of motion generation and control (33, 34). Hence, we believe that the increased ability of information conduction induced by glioma specifically implied patients with glioma would develop ISS during DCS. In addition, no differences in preoperative epileptic status and stimulation current were found between the ISS and non-ISS groups. Thus, we believe that the specific alterations in the sensorimotor network were more likely to be induced by glioma itself.

### Value of the Current Study

Duffau et al. (25) proposed that using ECoG was unnecessary. Because the low-intensity stimulation could not result in ISS, the absence of ECoG monitoring could simplify the surgical procedure. However, under the same stimulation protocol without ECoG monitoring, our patients experienced ISS [incidence was four times of that reported by Duffau (25)] with 1 or 1.5 mA as the initial stimulation current. Hence, we recommend that ECoG should not be omitted in some patients. Fortunately, the specific glioma-induced alterations in patients susceptible to ISS were found. These findings indicated that preoperative rs-fMRI may contribute to identifying patients who are more susceptible to ISS. For these susceptible patients, neurosurgeons should use ECoG to capture after-discharge current during DCS to avoid ISS.

## Conclusion

Patients with increased global and nodal efficiency, decreased shortest path length, and decreased FC induced by glioma are susceptible to ISS during DCS. In such patients, ECoG is recommended to monitor after-discharges during DCS to prevent ISS.

## Data Availability Statement

Anonymized data will be made available on request.

## Ethics Statement

The studies involving human participants were reviewed and approved by IRB of Beijing Tiantan Hospital. Written informed consent to participate in this study was provided by the participants' legal guardian/next of kin.

## Author Contributions

SF and CZ contributed to the study concept and design. SF, XF, YW, and ZZ contributed to data acquisition and analysis. SF, LW, ZZ, and YW contributed to the statistics/verified the analytical method. SF, CZ, and YW contributed to writing the first draft. XF, ZZ, YW, and TJ supervised the study, read and approved the final version. All authors contributed to the article and approved the submitted version.

## Conflict of Interest

The authors declare that the research was conducted in the absence of any commercial or financial relationships that could be construed as a potential conflict of interest.
