# Supplementary material for: Characteristic Alterations of Network in Patients With Intraoperative Stimulation-Induced Seizures During Awake Craniotomy
Source: Front Neurol. 2021 Mar 18;12:602716. doi: 10.3389/fneur.2021.602716 (PMC8012772; doi:10.3389/fneur.2021.602716)
Supplement: Supplementary file 1 [file Data_Sheet_1.DOCX]

# Part 1. Information of Topological Properties

# Global efficiency

Global efficiency means the ability of the information conduction in whole functional network. The calculation formula as follows (reference to supplementary materials of Ji G et al, Radiology, 2017([1](#_ENREF_1))):


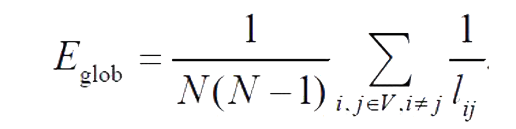


E*glob* = global efficiency of whole functional network; N = the number of actual edges connecting node *i* to other nodes in the whole functional network; l*i, k* = the shortest path length between the nodes j and k.

# Shortest path length

The shortest path length means that the consumption of minimum number of passing edges for information conduction. It describes the optimal path for information conduction from one node to another node in the whole network. The calculation formula as follows:


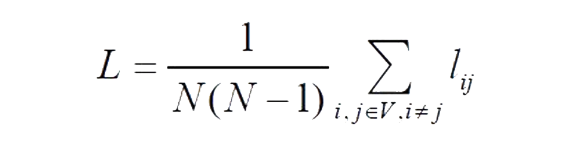


L = shortest path length of whole functional network; N = the number of actual edges connecting node *i* to other nodes in the whole functional network; l*i, k* = the shortest path length between the nodes j and k.

# Local efficiency

Local efficiency is an index to represent the ability of information conduction in local functional network. The calculation formula as follows:


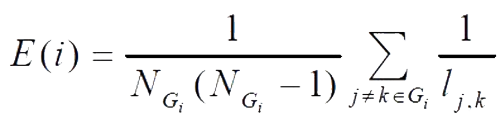


E*i* = local efficiency of whole functional network; NG*i* = the number of actual edges connecting node *i* to other nodes in the local functional network; l*i, k* = the shortest path length between the nodes j and k.

# Clustering coefficient

Cluster coefficient is an important index to measure the degree of clustering of the functional network, which represents the possibility that the neighbors of node *i* can interact with other nodes. The calculation formula as follows:

$$Ci=\frac{2ei}{ki(ki-1)}$$

C*i* = cluster coefficient of node *i*; e*i* = the number of actual edges connecting to other nodes; k*i* = the number of probable edges connecting to other nodes.

# Nodal efficiency


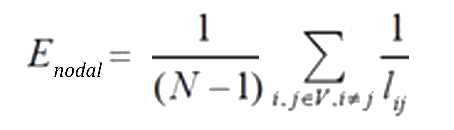
 Nodal efficiency means the ability of the information conduction of the node i. The calculation formula as follows:

E*nodal* = nodal efficiency of whole functional network; N = the number of actual edges connecting node *i* to other nodes in the whole functional network; l*i, k* = the shortest path length between the nodes j and k.

# Small-worldness properties

Small-worldness properties, including gamma, lambda, and sigma, represent the efficiency of information delivery; gamma (γ) = C_real_/C_random_ >> 1 (C represented cluster coefficient), lambda (λ) = L_real_/L_random_ ~ 1 (L represented shortest path length), and sigma (σ) = γ/λ > 1.([2](#_ENREF_2), [3](#_ENREF_3)) A high value of sigma represents a high efficiency of information delivery.

# Part 2. Supplemental Tables

**Table S1. Montreal Neurological Institute (MNI) locations of 22 nodes in the sensorimotor network**

| **Regions of interesting** | **Modified Cyto-architectonic** | **Left hemisphere** | | | **Right hemisphere** | | |
| --- | --- | --- | --- | --- | --- | --- | --- |
|  |  | X | Y | Z | X | Y | Z |
| A6m_L(R) | Medial area BA 6 | 5 | 36 | 38 | 6 | 38 | 35 |
| A4hf_R | Area BA 4 (head and face) | - | - | - | 55 | -2 | 33 |
| A4ul_L(R) | Area BA 4 (upper limb) | -26 | -25 | 63 | 34 | -19 | 59 |
| A4t_L(R) | Area BA 4 (trunk) | -13 | -20 | 73 | 15 | -22 | 71 |
| A4tl_R | Area BA 4 (tongue and larynx) | - | - | - | 54 | 4 | 9 |
| A6cvl_L(R) | caudal ventrolateral Area BA 6 | -49 | 5 | 30 | 51 | 7 | 30 |
| A1_2_3ll_L(R) | Area BA 1/2/3 (lower limb) | -8 | -38 | 58 | 10 | -34 | 54 |
| A4ll_L(R) | Area BA 4 (lower limb) | -4 | -23 | 61 | 5 | -21 | 61 |
| A1_2_3ulhf_L(R) | Area BA 1/2/3 (upper limb and face) | -50 | -16 | 43 | 50 | -14 | 44 |
| A1_2_3tonIa_L(R) | Area BA 1/2/3 (tongue and larynx) | -56 | -14 | 16 | 56 | -10 | 15 |
| A2_L(R) | Area BA 2 | -46 | -30 | 50 | 48 | -24 | 48 |
| A1_2_3tru_L(R) | Area BA 1/2/3 (trunk) | -21 | -35 | 68 | 20 | -33 | 69 |

*****BA = Brodmann area.

**Table S2. Functional connections with significant differences among the ISE, non-ISE, and health groups**

| **Connections** | **Functional connectivity (mean ± SEM)** | | | ***p* value**  (ISE vs non-ISE) | ***p* value**  (ISE vs healthy) | ***p* value**  (non-ISE vs healthy) |
| --- | --- | --- | --- | --- | --- | --- |
|  | ISE group | non-ISE group | Health group |  |  |  |
| Am6_R and A4hf_R | 0.245 ± 0.064 | 0.689 ± 0.066 | 0.343 ± 0.035 | 0.0002 | 0.1666 | 0.0001 |
| A4t_R and A4hf_R | 0.515 ± 0.089 | 0.861 ± 0.084 | 0.417 ± 0.071 | 0.0007 | 0.7412 | 0.0001 |
| A4ll_R and A4hf_R | 0.297 ± 0.057 | 0.613 ± 0.048 | 0.284 ± 0.043 | 0.0008 | 0.8587 | 0.0001 |
| A4ul_R and A1_2_3tonIa_L | 0.173 ± 0.069 | 0.618 ± 0.076 | 0.242 ± 0.063 | 0.0007 | 0.5147 | 0.0016 |
| A4ul_L and A1_2_3tonIa_L | 0.240 ± 0.125 | 0.452 ± 0.098 | 0.312 ± 0.045 | 0.1930 | 0.0066 | 0.0096 |
| A4ll_R and A6m_R | 0.515 ± 0.089 | 0.861 ± 0.084 | 0.417 ± 0.071 | 0.0154 | 0.4359 | 0.0010 |
| A4ll_L and A4hf_R | 0.299 ± 0.056 | 0.660 ± 0.078 | 0.279 ± 0.041 | 0.0022 | 0.7841 | 0.0001 |
| A1/2/3tru_L and A4hf_R | 0.256 ± 0.070 | 0.616 ± 0.079 | 0.320 ± 0.033 | 0.0045 | 0.3673 | 0.0005 |
| A4ul_R and A4ll_R | 0.486 ± 0.097 | 0.821 ± 0.049 | 0.532 ± 0.037 | 0.0093 | 0.5147 | 0.0001 |
| A4t_R and A1_2_3tonIa_R | 0.274 ± 0.071 | 0.639 ± 0.070 | 0.210 ± 0.062 | 0.0027 | 0.5473 | 0.0003 |
| A4ll_L and A1_2_3tonIa_R | 0.310 ± 0.063 | 0.587 ± 0.067 | 0.289 ± 0.050 | 0.0101 | 0.8060 | 0.0020 |
| A4ll_R and A1_2_3tonIa_R | 0.392 ± 0.064 | 0.630 ± 0.072 | 0.297 ± 0.057 | 0.0312 | 0.3275 | 0.0021 |

***** BA = Brodmann area. Am6_R = medial BA 6 in the right hemisphere; A4hf_R = head and face region of BA 4 in the right hemisphere; A4ll_L = lower limb region of BA 4 in the right hemisphere; A4ll_R = lower limb region of BA 4 in the right hemisphere; A4ul_R = upper limb region of BA 4 in the right hemisphere; A4t_R = trunk region of BA 4 in the right hemisphere; A1/2/3tru_L = trunk region of BA 1/2/3 in the left hemisphere; A1/2/3tonIa_L = tongue and larynx region of BA 1/2/3 in the left hemisphere; A1/2/3tonIa_R = tongue and larynx region of BA 1/2/3 in the right hemisphere. Threshold of false discover rate correction was p value = 0.0021.

**Table S3. Topological properties compared between the patient and healthy groups**

|  | ISE group | non-ISE group | Health group | One-way ANOVA  (p value) | Post-hoc analysis  (p value) | | |
| --- | --- | --- | --- | --- | --- | --- | --- |
|  |  |  |  |  | ISE vs non-ISE | ISE vs Health | non-ISE vs Health |
| Global efficiency | 0.651 ± 0.002 | 0.636 ± 0.004 | 0.646 ± 0.004 | 0.041 | 0.027 | 0.425 | 0.072 |
| Local efficiency | 0.717 ± 0.023 | 0.667 ± 0.013 | 0.740 ± 0.011 | 0.009 | 0.060 | 0.309 | 0.002 |
| Shortest path length | 1.850 ± 0.013 | 1.966 ± 0.034 | 1.892 ± 0.029 | 0.043 | 0.026 | 0.337 | 0.095 |
| Clustering coefficient | 0.685 ± 0.020 | 0.680 ± 0.0112 | 0.653 ± 0.011 | 0.196 | - | - | - |
| Gamma | 1.195 ± 0.062 | 1.075 ± 0.030 | 1.249 ± 0.038 | 0.037 | 0.116 | 0.416 | 0.011 |
| Lambda | 1.062 ± 0.006 | 1.116 ± 0.016 | 1.101 ± 0.014 | 0.066 | - | - | - |
| Sigma | 1.127 ± 0.059 | 0.968 ± 0.038 | 1.14 ± 0.041 | 0.046 | 0.044 | 0.839 | 0.017 |

***** The global properties were calculated with one-way ANOVA test. If the results one-way ANOVA were significance, post-hoc analysis with least significant difference was subsequently applied.

**Table S4. Nodal efficiency compared between the patients and healthy groups**

| Node name | ISE group | non-ISE group | Health group | One-way ANOVA  (p value) | Post-hoc analysis  (p value) | | |
| --- | --- | --- | --- | --- | --- | --- | --- |
|  |  |  |  |  | ISE vs non-ISE | ISE vs non-ISE | non-ISE vs Health |
| A6m_L | 0.588 ± 0.067 | 0.412 ± 0.089 | 0.514 ± 0.050 | 0.286 | - | - | - |
| A6m_R | 0.542 ± 0.064 | 0.611 ± 0.043 | 0.620 ± 0.038 | 0.516 | - | - | - |
| A4hf_R | 0.509 ± 0.059 | 0.507 ± 0.061 | 0.532 ± 0.034 | 0.913 | - | - | - |
| A4ul_L | 0.589 ± 0.046 | 0.610 ± 0.067 | 0.651 ± 0.023 | 0.554 | - | - | - |
| A4ul_R | 0.647 ± 0.036 | 0.581 ± 0.021 | 0.617 ± 0.022 | 0.333 | - | - | - |
| A4t_L | 0.597 ± 0.036 | 0.484 ± 0.072 | 0.569 ± 0.037 | 0.339 | - | - | - |
| A4t_R | 0.629 ± 0.025 | 0.597 ± 0.031 | 0.581 ± 0.025 | 0.500 | - | - | - |
| A4tl_R | 0.466 ± 0.054 | 0.182 ± 0.071 | 0.437 ± 0.052 | 0.009 | 0.007 | 0.740 | 0.006 |
| A6cvl_L | 0.258 ± 0.064 | 0.250 ± 0.074 | 0.369 ± 0.054 | 0.329 | - | - | - |
| A6cvl_R | 0.392 ± 0.079 | 0.208 ± 0.075 | 0.431 ±0.049 | 0.067 | - | - | - |
| A1_2_3ll_L | 0.594 ± 0.024 | 0.457 ± 0.084 | 0.629 ± 0.018 | 0.022 | 0.055 | 0.561 | 0.007 |
| A1_2_3ll_R | 0.596 ± 0.028 | 0.543 ± 0.034 | 0.581 ± 0.024 | 0.517 | - | - | - |
| A4ll_L | 0.546 ± 0.061 | 0.593 ± 0.065 | 0.627 ± 0.020 | 0.439 | - | - | - |
| A4ll_R | 0.652 ± 0.023 | 0.624 ± 0.032 | 0.649 ± 0.024 | 0.778 | - | - | - |
| A1_2_3ulhf_L | 0.578 ± 0.073 | 0.561 ± 0.063 | 0.654 ± 0.021 | 0.305 | - | - | - |
| A1_2_3ulhf_R | 0.675 ± 0.034 | 0.585 ± 0.036 | 0.662 ± 0.017 | 0.090 | - | - | - |
| A1_2_3tonIa_L | 0.395 ± 0.073 | 0.498 ± 0.055 | 0.539 ± 0.031 | 0.135 | - | - | - |
| A1_2_3tonIa_R | 0.574 ± 0.028 | 0.508 ± 0.071 | 0.566 ± 0.033 | 0.607 | - | - | - |
| A2_L | 0.563 ± 0.067 | 0.310 ± 0.083 | 0.549 ± 0.025 | 0.006 | 0.006 | 0.854 | 0.003 |
| A2_R | 0.553 ± 0.048 | 0.531 ± 0.036 | 0.571 ± 0.022 | 0.704 | - | - | - |
| A1_2_3tru_L | 0.692 ± 0.015 | 0.572 ± 0.064 | 0.675 ± 0.014 | 0.049 | 0.028 | 0.712 | 0.029 |
| A1_2_3tru_R | 0.683 ± 0.012 | 0.601 ± 0.065 | 0.696 ± 0.015 | 0.126 | - | - | - |

***** The global properties were calculated with one-way ANOVA test. If the results one-way ANOVA were significance, post-hoc analysis with least significant difference was subsequently applied.

# Part 3. Supplemental Figures


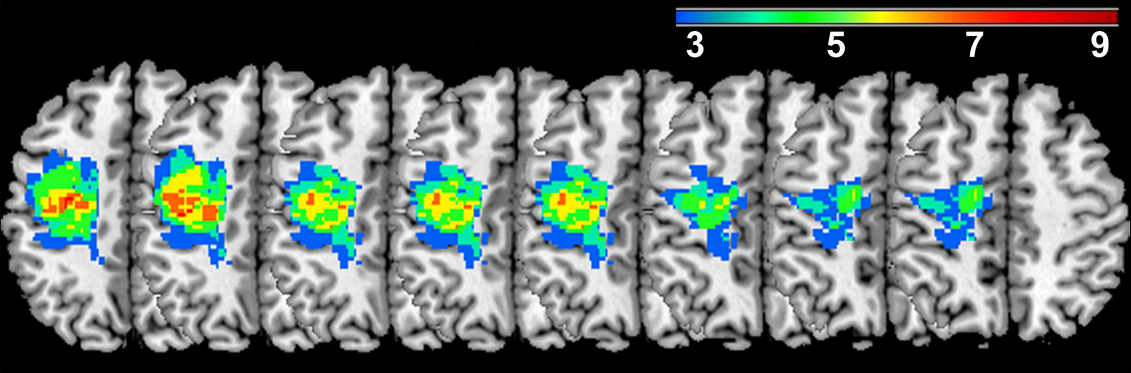


**Figure. S1.** The overlapping results of prefrontal lobe gliomas in the left hemisphere. The value of color bar represents the number of patients with tumor located in a same region.


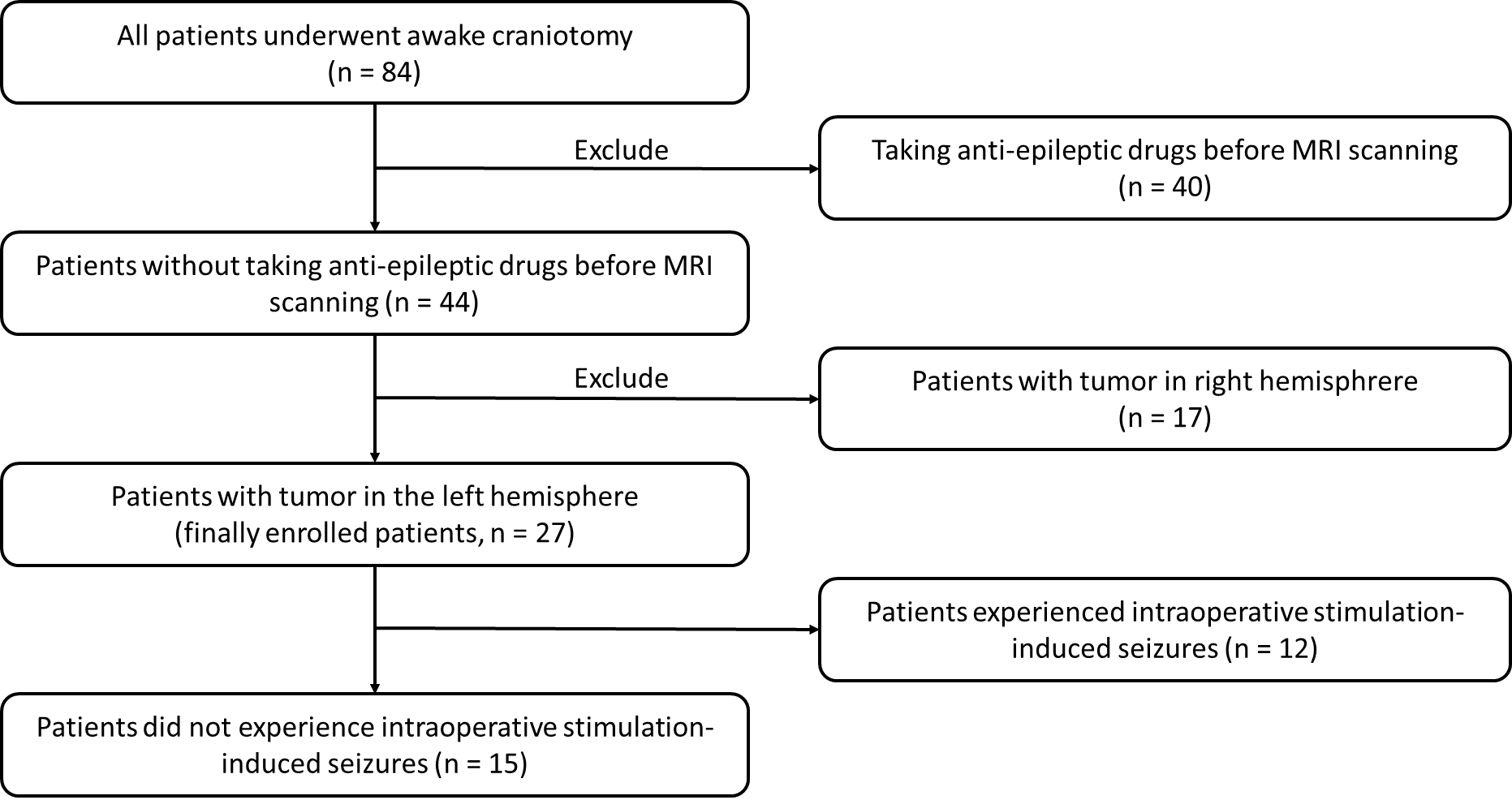
 **Figure. S2.** The step of patient selection.

# Reference

1. G. J. Ji, Y. Yu, H. H. Miao, Z. J. Wang, Y. L. Tang and W. Liao: Decreased Network Efficiency in Benign Epilepsy with Centrotemporal Spikes. *Radiology*, 283(1), 186-194 (2017) doi:10.1148/radiol.2016160422

2. M. D. Humphries, K. Gurney and T. J. Prescott: The brainstem reticular formation is a small-world, not scale-free, network. *Proc Biol Sci*, 273(1585), 503-11 (2006) doi:10.1098/rspb.2005.3354

3. Y. Gong, H. Wu, J. Li, N. Wang, H. Liu and X. Tang: Multi-Granularity Whole-Brain Segmentation Based Functional Network Analysis Using Resting-State fMRI. *Front Neurosci*, 12, 942 (2018) doi:10.3389/fnins.2018.00942
